# Overview and Recent Progress on the Biosynthesis and Regulation of Flavonoids in *Ginkgo biloba* L.

**DOI:** 10.3390/ijms241914604

**Published:** 2023-09-27

**Authors:** Jing Guo, Yeqiao Wang, Jiaqi Li, Jingjing Zhang, Yaqiong Wu, Guibin Wang

**Affiliations:** 1 State Key Laboratory of Tree Genetics and Breeding, Co-Innovation Center for Sustainable Forestry in Southern China, Nanjing Forestry University, 159 Longpan Road, Nanjing 210037, China; jingguo@njfu.edu.cn (J.G.); wyq8513@163.com (Y.W.); ljq2015@njfu.edu.cn (J.L.); star77t@163.com (J.Z.); 2 Institute of Botany, Jiangsu Province and Chinese Academy of Sciences (Nanjing Botanical Garden Mem. Sun Yat-Sen), Qian Hu Hou Cun No. 1, Nanjing 210014, China; ya_qiong@126.com

**Keywords:** ginkgo, flavonoids, biosynthetic pathways, transcriptional regulation, influencing factors

## Abstract

Flavonoids and their derivatives play important roles in plants, such as exerting protective activity against biotic and abiotic stresses, functioning in visual signaling to attract pollinators, and regulating phytohormone activity. They are also important secondary metabolites that are beneficial to humans. *Ginkgo biloba* L. is a well-known relict plant considered to be a “living fossil”. Flavonoids present in ginkgo leaves have antioxidant and anti-aging capacities and show good therapeutic effects on a variety of neurological diseases. To date, studies on flavonoids have mainly focused on their extraction, pharmacological effects, and component analysis and on the expression levels of the key genes involved. However, a systematic review summarizing the biosynthesis and regulatory mechanisms of ginkgo flavonoids is still lacking. Thus, this review was conducted to comprehensively introduce the biological characteristics, value, and utilization status of ginkgo; summarize the effects, biosynthetic pathways, and transcriptional regulation of flavonoids; and finally, discuss the factors (ecological factors, hormones, etc.) that regulate the biosynthesis of flavonoids in ginkgo. This review will provide a reference basis for future research on the biosynthesis and efficient utilization of flavonoids in ginkgo.

## 1. Introduction

Plants have been used to treat diseases for thousands of years, and there are currently many safe and effective health products and medicines made from plant-based ingredients on the market; a large proportion of these ingredients are secondary metabolites [1,2]. *Ginkgo biloba* L. (ginkgo) is the oldest relict plant among the existing gymnosperms and is the only remaining species of Ginkgoaceae in Ginkgoales. Ginkgo survived the Quaternary glaciation and has developed increased resistance by synthesizing a variety of secondary metabolites, making it highly adaptable to harsh environments during the long evolutionary process [3,4]. These secondary metabolites confer ginkgo with important medicinal value; for instance, the abundant flavonoids, terpene lactones, and other active substances present in ginkgo leaves have made ginkgo leaf extract a focus of attention in the herbal medicine industry. To date, the exploitation of ginkgo has mainly focused on using ginkgo leaf extract to develop health care and pharmaceutical products with good therapeutic effects on heart disease, coronary heart disease, senile dementia, nervous system diseases and many other chronic and acute diseases [5,6,7,8]. The current main method for obtaining active substances is still to extract them from ginkgo leaves. Therefore, effectively increasing the content of active substances in ginkgo leaves is a topic in urgent need of research.

There have been many reviews of ginkgo, and each has its own unique characteristics and highlights. For example, Cheng et al. [9] summarized research progress on understanding regulatory factors and ecological factors affecting flavonoid concentrations and the biotechnological and chemical synthesis techniques utilized to produce flavonoids. Liu et al. [10] summarized domestic and international research progress on the chemical composition analysis, sample preparation, separation, detection, and different quality standards of flavonoids in ginkgo leaves and their finished products from 2009 to 2014 and comprehensively compared the advantages and disadvantages of various analytical methods and their chromatographic conditions. In recent years, scholars from related disciplines have summarized and comparatively analyzed the chemical composition of ginkgo leaves and seeds as well as methods for their extraction, purification, and analysis [11,12]. The physical and chemical properties, biological activities, and allergenic glycoproteins of ginkgo kernel proteins have been systematically analyzed and the related advantages and disadvantages evaluated, providing prospects for future research directions and suggestions for future research [12,13]. Li et al. [14] summarized the practical significance and application of an extraction method with significant advantages, such as the supercritical carbon dioxide fluid extraction (SFE-CO_2_) method, which can be employed in the extraction and separation of active ingredients in ginkgo leaves, seeds, pollen, and roots to make full use of ginkgo’s resources. Finally, the effects on cardiovascular activity and potential adverse reactions to ginkgo leaf extract were summarized, and the safety of its potential clinical application was also demonstrated [6].

In general, several aspects of ginkgo utilization have been summarized, but the resolution of the biosynthesis pathway of ginkgo leaf flavonoids and methods for content enhancement remain to be reported, and strategies for maintaining stable growth with higher quality and enhancing the efficiency of ginkgo leaf yields still need careful consideration. Currently, with the rapid development of omics research in systems biology, emergent transcriptome, metabolome, and proteome studies related to the biosynthesis and metabolism of flavonoids in ginkgo leaves provide an opportunity to explore the mechanism of their metabolic synthesis. However, it has been found that single omics analyses cannot address the metabolic regulation problem well, so an integrated multiple-omics analysis was performed to reflect changes in gene and protein expression levels and metabolite abundance in ginkgo flavonoid biosynthesis pathways, providing new insights for exploring its metabolic mechanism.

It is effective to use metabolic engineering strategies to improve the biosynthesis of ginkgo flavonoids by mining biosynthetic genes and clarifying transcriptional regulatory mechanisms. Accordingly, this review focuses on providing a systematic and comprehensive overview of ginkgo flavonoid secondary metabolites and their metabolic pathways. The specific objectives are to (1) elaborate the biological characteristics, value, and utilization status of ginkgo; (2) review the main components and functions of flavonoids in ginkgo leaves; (3) summarize flavonoid biosynthesis pathways and related transcriptional regulatory factors; (4) conduct an in-depth analysis of ecological factors and hormones that affect flavonoid biosynthesis; and finally, (5) discuss the limitations and development directions of ginkgo flavonoid research and utilization in the hope of providing a reference for future research on secondary metabolites such as flavonoids in ginkgo.

## 2. Biological Characteristics of Ginkgo

Ginkgo is the oldest relict plant among the existing gymnosperms and is commonly known as a “living fossil”; it is also called the Gongsun tree, duck foot tree, maidenhair tree or white fruit tree [15,16]. Ginkgo trees are tall with straight trunks, and some ancient ginkgo trees in China are over 50 m in height and 4.6 m in diameter at breast height. Ginkgo trees have a long lifespan, and many ginkgo trees in China are over 1000 years old. The ancient ginkgo at Dinglin Temple in Fulai Mountain, Ju County, Shandong Province, is more than 3000 years old and still bears fruit [17]. Ginkgo leaves have a unique fan shape and turn bright yellow in late autumn, giving trees high ornamental value. Ginkgo is a dioecious, unisexual tree species, and its flowers are wind-pollinated. The seeds are often elliptic, and their fleshy outer layer (the exotesta) is light yellow-brown, soft, fruit-like, and covered with white powder, and it emits an unpleasant smell after falling.

Ginkgo was widely distributed worldwide before the Quaternary glaciation but was preserved only in China after this period, and the Jurassic (in the Mesozoic) was the most prosperous period for the development of Ginkgoopsida, with at least 16 genera [4,18]. Ginkgo is the only species of Ginkgoopsida that has survived to modern day and is now widely cultivated in temperate and subtropical regions worldwide due to its high ornamental value and significant environmental adaptability [19]. It has long been believed that ginkgo trees planted in Europe, Japan, Korea, and America have been dispersed multiple times from Eastern China [18]. Recent research also suggests that ginkgo trees found worldwide originated from populations in eastern China and confirms the important role of human intervention in the migration of ginkgo from its refuges to other parts of China and to the world [4].

The unique morphological and structural characteristics and phylogenetic and taxonomic classification status of ginkgo and its important scientific research value have been emphasized by academics [17]. A draft genome of the living fossil *Ginkgo biloba* was completed in 2016 based on next-generation sequencing, and the assembled genome is approximately 10.61 Gb in size [20]. A combination of second- and third-generation sequencing methods were employed to complete ginkgo genome sequencing, construct a 9.87 Gb genome sequence, and obtain 27,834 genes, 91% of which were functionally annotated. The completion of the assembly of high-quality whole-genome sequences provides a basis for biological studies of ginkgo and other gymnosperms and provides useful genetic resources for studying their important gene functions.

## 3. The Value and Utilization Status of Ginkgo

**Figure 1:**
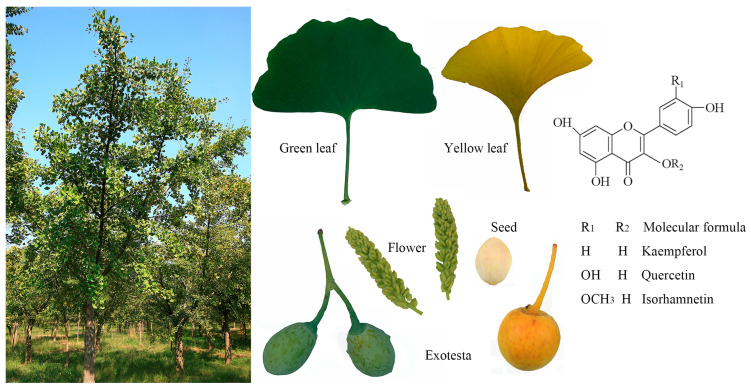
Phenotype and three main single flavonoid structures of ginkgo.

Ginkgo is a treasure tree species that has multiple ecological and economic purposes and is valuable for food, medicine, timber, landscaping, and ecological protection [21]. The well-known Chinese medical texts Compendium of Materia Medica and Benjing Fengyuan record the use of ginkgo leaves in traditional Chinese medicine for phlegm resolution, detoxification, and the treatment of diarrhea and frequent urination [22]. Moreover, according to the new edition of the Chinese Pharmacopoeia, ginkgo leaves and preparations thereof are permitted for market sale, and the listed indications include activating blood circulation and removing blood stasis [22]. Flavonoids, terpenoids, ginkgolic acids, phenols, and polysaccharides are common chemical components that have a wide range of biological activities in ginkgo leaves, seeds, and exotesta ([22], Figure 1). Specifically, ginkgo seeds, commonly known as white fruits, are rich in carbohydrates, protein, and fat, show high nutritional value, and are often used in the preparation of Chinese cuisine [23,24]. However, ginkgo kernels contain slightly toxic substances, such as ginkgolic acid, 4’-O-methylpyridoxine (MPN), and its glucoside, and the dosage should be carefully controlled [12]. Ginkgo leaves are rich in various complex chemical components, with over 180 reported components, including active components such as flavonoids, terpenoids, polyphenols, and amino acids [5]. In addition, they include polysaccharides, phenolic acids, steroid compounds, and trace elements. The specifications for ginkgo leaf extract all require a flavonoid content higher than 24%, a ginkgolide content higher than 6%, and a ginkgolic acid content of less than 5 ppm. Flavonoids are the most important components of ginkgo leaf extract and can be divided into flavonoids and flavonoid glycosides, biflavonoids, catechins, and other major categories according to structure. Among flavonoid glycosides, quercetin, kaempferol, and isorhamnetin are the most relevant structures ([25]; Figure 1). Currently, the total production of standardized extracts of ginkgo leaves (*Ginkgo biloba* extract 761, GBE761) exceeds 3 million tons, accounting for approximately 1/6 of the total demand in the international market, which has led to an immense industry of ginkgo leaf preparations [26]. The annual sales of health food and pharmaceutical products developed from ginkgo have exceeded $2 billion [27,28]. Therefore, selecting ginkgo varieties with high flavonoid contents and increasing leaf flavonoid contents have become key goals in the efficient cultivation and genetic breeding of ginkgo.

## 4. Introduction and Function of Flavonoids in Ginkgo

Flavonoids are an important class of natural secondary metabolites found in ferns, mosses, and seed plants (gymnosperms and angiosperms) and usually accumulate in the form of glycosides in the vacuoles of plant cells. They are a class of low-molecular-weight polyphenolic compounds with a C6-C3-C6 backbone [2]. According to the degree of oxidation of the C3 structure and the position of the B-ring connection, flavonoids can usually be divided into flavones, flavonols, isoflavones, flavanones, flavanols, anthocyanidins, and chalcones [2,29]. A total of 110 flavonoids belonging to these seven classes with unambiguous structures have been reported, many of which are detected in ginkgo leaves [11]. Moreover, 13 biflavonoids have been identified from ginkgo leaves, including amentoflavone, bilobetin, sciadopitysin, ginkgetin, and isoginkgetin, which are the most common [30]. In LC–MS-based nontarget metabolomics, mainly conducted through the unbiased detection of all small-molecule metabolites, many flavonoid metabolites have been identified, and differences between treatments have also been observed [5]. Further targeted metabolomics analyses focused on enriched flavonoids need to be conducted to study their contents [31].

Liu et al. [32] identified 13 gene families in the ginkgo flavonoid biosynthetic pathway and obtained 111 enzyme-encoding genes based on ginkgo genome information. These genes have high copy numbers, and multiple genes contain large introns. The promoters of 111 enzyme-encoding genes contain light-responsive elements as well as various hormone-related and stress-responsive elements. To date, studies on the molecular mechanisms of ginkgo flavonoid metabolism have focused on both structural and regulatory genes in the flavonoid biosynthetic pathway [5,33,34,35].

## 5. Biosynthetic Pathways and Transcriptional Regulation

### 5.1. Biosynthetic Pathways

**Figure 2:**
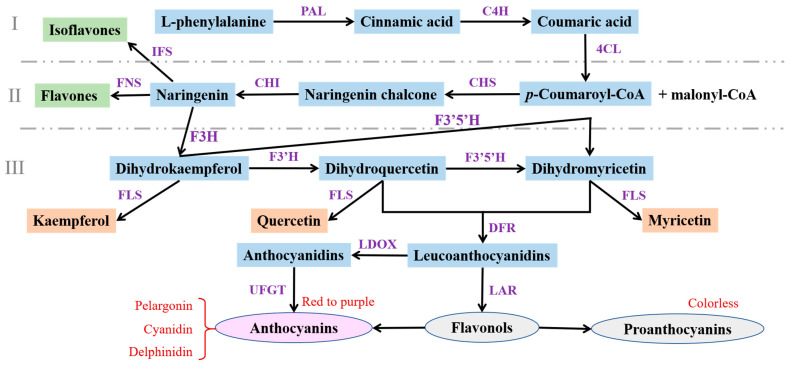
Schematic diagram of the ginkgo flavonoid biosynthesis pathway. (**I**) The initial reaction of flavonoid metabolism, (**II**) The key reaction in the biosynthesis of ginkgo flavonoids, (**III**) The synthesis of various flavonoids and anthocyanins. The pathway involves key structural enzyme-coding genes and intermediate metabolites. PAL: phenylalanine ammonia lyase; C4H: cinnamate-4-hydroxylase; 4CL: 4-coumaroyl-CoA ligase; CHS: chalcone synthase; CHI: chalcone isomerase; IFS: 2-hydroxyflavonone synthase; FNS: flavone synthase; F3H: flavanone 3-hydroxylase; F3′H: flavonol 3′-hydroxylase; F3′5′H: flavonol 3′5′-hydroxylase; FLS: flavonol synthase; DFR: dihydroflavonol 4-reductase; LDOX: leucoanthocyanidin dioxygenase; LAR: leucoanthocyanidin reductase; UFGT: flavonoid-3-*O*-glycosyltransferase. The orange box represents three types of flavonols.

The biosynthesis of flavonoids as active ingredients in ginkgo begins with the common precursor substance L-phenylalanine, which is derived from the mangiferylic acid pathway. Flavonoids are mainly produced through the phenylalanine metabolic pathway, which can basically be divided into three stages ([36], Figure 2). The first stage is the initial reaction of flavonoid metabolism, from phenylalanine to the *p*-coumaroyl-CoA stage, which begins with catalysis of phenylalanine aminolytic enzyme (PAL) and the formation of *p*-coumaroyl-CoA through the action of cinnamic acid 4-hydroxylase (C4H) and 4-coumaroyl-CoA ligase (4CL) enzymes.

The second stage is the key reaction in the biosynthesis of ginkgo flavonoids, from *p*-coumaroyl-CoA and malonyl-CoA as substrates to the dihydroflavonol stage (Figure 2). Using *p*-coumaroyl-CoA and malonyl-CoA as starting substrates in the synthetic pathway, chalcone synthase (CHS) catalyzes the formation of naringenin chalcone. Naringenin chalcone is then catalyzed by chalcone isomerase (CHI) to form naringenin, which can also be produced spontaneously without enzyme (Figure 2). Naringenin enters the synthetic pathway of other flavonoid compounds.

The third stage is the synthesis of various flavonoids and anthocyanins, from dihydroflavonols to various anthocyanins (Figure 2). Naringenin can form not only isoflavones and flavonoids through the action of 2-hydroxyflavonone synthase (IFS) and flavone synthase (FNS), respectively, but also dihydroflavonols under the action of flavanone 3-hydroxylase (F3H). Subsequently, dihydroflavonols are subject to the action of flavonol synthase (FLS), forming flavonols, including kaempferol, quercetin, and myricetin, which are then subject to the action of dihydroflavonol 4-reductase (DFR), forming leucoanthocyanins. Leucoanthocyanidins can be transformed to colorless flavanols, catalyzed by leucoanthocyanidin reductase (LAR), or anthocyanidins, catalyzed by leucoanthocyanidin dioxygenase (LDOX). Anthocyanidins undergo glycosylation mediated by flavonoid-3-*O*-glycosyltransferase (UFGT) to form anthocyanins, which remain stable in ginkgo leaves and may undergo different modifications, such as glycosylation, methylation, and acylation, to form different types of anthocyanins (Figure 2).

### 5.2. Structural Enzyme Genes

Structural genes are key enzyme-coding genes in the biosynthesis pathway of flavonoids that directly regulate biosynthesis. A variety of key structural genes have been identified in ginkgo, and their functions have been explored via in vitro assays of enzyme activities and transgenic assays (Table 1). PAL from *G. biloba* (GbPAL), the first key enzyme in flavonoid metabolism, is constitutively expressed in all tissues of ginkgo, with higher expression in leaves and stems. The transcript levels of GbPAL are significantly correlated with flavonoid accumulation, indicating that GbPAL may play a regulatory role in the biosynthesis of flavonoids at the transcriptional level [37]. An enzymatic assay revealed that a recombinant C4H protein from *G. biloba* (GbC4H) catalyzes the conversion of trans-cinnamic acid to *p*-coumaric acid [38]. Previous studies revealed that GbC4H is highly expressed in stems and roots, while low expression levels are observed in seeds, seed-pedicels, and petioles. The lignin content is positively correlated with the level of GbC4H transcripts in different tissues. The transcription levels of GbC4H are increased under UV-B, cold, salicylic acid, and abscisic acid treatments, indicating that GbC4H may play a role in stress and hormonal signaling responses. 4CL not only provides the precursor substance 4-coumaroyl-CoA for chalcone synthesis but also regulates plant lignin formation and cell differentiation. 4CL activity shows different trends under different combinations of diurnal temperatures [39].

The first CHS from *G. biloba* (GbCHS) was cloned in 2004, and its sequence was found to be highly homologous to those from other gymnosperms [40]. CHI is a key gene that regulates the accumulation of total flavonoids in ginkgo, and its expression has tissue specificity [41]. HPLC assays of in vitro enzyme activity showed that a recombinant CHI protein from *G. biloba* (GbCHI) catalyzes the formation of naringin from 6’-hydroxychalcone. CHI activity is correlated with the levels of CHI gene transcription, and GbCHI activity is positively correlated with total flavonoid levels in ginkgo leaves [41]. The activity of F3H is also positively correlated with the biosynthesis of flavonoids, and the expression levels of the F3H gene from *G. biloba* (GbF3H) are higher in stems and leaves, with the highest levels in leaves [42]. F3′H and F3′5′H belong to the cytochrome P450 superfamily: because a transgenic system has not yet been established in ginkgo, the heterologous overexpression of F3′H was conducted and was shown to increase epigallocatechin, gallocatechin, and catechin contents, while F3′5′H overexpression increases epicatechin and gallocatechin contents [33,34,35].

A recombinant FLS protein from *G. biloba* (GbFLS) has been shown to catalyze the transformation of dihydrokaempferol to kaempferol and the transformation of kaempferol to naringenin, indicating that GbFLS is a bifunctional enzyme in the flavonoid biosynthetic pathway [43]. Recent research suggests that GbFLSa negatively regulates proanthocyanidin biosynthesis [44]. Two dihydroflavonol 4-reductase proteins from *G. biloba* (GbDFR1 and GbDFR3) both catalyze the conversion of dihydroquercetin to anthocyanins, while GbDFR2 catalyzes the conversion of dihydrophorbol to anthocyanins [45]. The overexpression of GbDFR6 changes the flowering phenotype under short-day conditions and increases the contents of many anthocyanins [47]. Additionally, the overexpression of GbDFR6 in ginkgo leads to a self-incompatibility-like phenotype in transgenic tobacco [48].

### 5.3. Transcriptional Regulation

Structural genes are regulated by regulatory factors and have specific spatiotemporal regulatory mechanisms, resulting in the formation of different metabolites [49]. Transcription factors, miRNAs, and lncRNAs can regulate the expression of structural genes. The top-ranked miRNA target genes are associated with plant pathogen interactions, plant hormone signaling, and flavonoid biosynthesis [50]. Liu et al. [51] demonstrated significant enrichment of cis-regulatory target genes of upregulated lncRNAs in flavonoid biosynthetic pathways, and lncRNAs may serve as precursors and endogenous targeting mimics for miRNAs, indirectly regulating protein-coding genes. More importantly, multiple lncRNAs may act as targets of miR156a, miR172a, miR396a, miR828a, and miR858a and then participate in the synthesis of ginkgo flavonoids by forming a lncRNA (target)–miRNA–PCene network [51]. It is thus clear that lncRNAs may be involved in the regulation of ginkgo flavonoid biosynthesis in multiple ways.

Transcription factors (TFs) can compensate for the lack of activity of single key enzyme-encoding genes and the possible constitutive lethal expression of multiple enzyme-encoding genes in metabolic engineering via “multipoint regulation” [52]. TFs such as R2R3-MYB, bHLH, and WD40 proteins play an important role in gene expression and regulation in ginkgo and can function independently or coordinate with other factors to control multiple enzyme steps involved in flavonoid biosynthesis pathways [53]. Yang et al. [54] found 69 R2R3-MYB family members in ginkgo, and synaptic analysis suggested that a few tandem and segmental duplications may lead to contraction of the GbR2R3-MYB gene family. GbR2R3-MYB gene family members show distinct spatiotemporal expression patterns, and many of them have been isolated and characterized; for example, GbMYBF2 and GbMYBFL have been proven to be negatively and positively correlated with flavonoid contents, respectively. Their expression can be induced by abiotic stresses or hormones [55,56]. In addition, GbMYBR1 (A novel type of R2R3 MYB repressor, desgnited by Su et al. [57]) has pleiotropic effects on plant growth, phenylpropanoid accumulation, and trichome development mediated by interaction with Glabrous 3 (GL3) or the direct repression of key pathway genes. Zhou et al. [58] employed genomic and transcriptomic databases to identify GbbHLH gene family members, and 85 GbbHLH genes belonging to 17 subfamilies were identified, among which 7 genes were screened for potential involvement in flavonoid biosynthesis. A total of 167 WD40 family members were identified in ginkgo, which were divided into 5 clusters and 16 subfamilies. Promoter analysis showed that five GbWD40 genes had structural sites involved in flavonoid metabolism regulation in their promoter regions, and further correlation analysis identified 6 GbWD40 genes that may be involved in flavonoid metabolism [59]. The overexpression of GbLWD1, a WD40 family member, significantly promoted the synthesis of flavonoids in transgenic poplar, thereby improving the salt tolerance of poplar [60]. A total of 40 bZIP family members were identified and classified into 10 subfamilies in ginkgo, and further correlation analysis and phylogenetic tree analysis indicated that GbbZIP08 and GbbZIP15 might be involved in the biosynthesis of flavonoids [61].

## 6. Factors Regulating the Synthesis and Metabolism of Ginkgo Flavonoids

**Figure 3:**
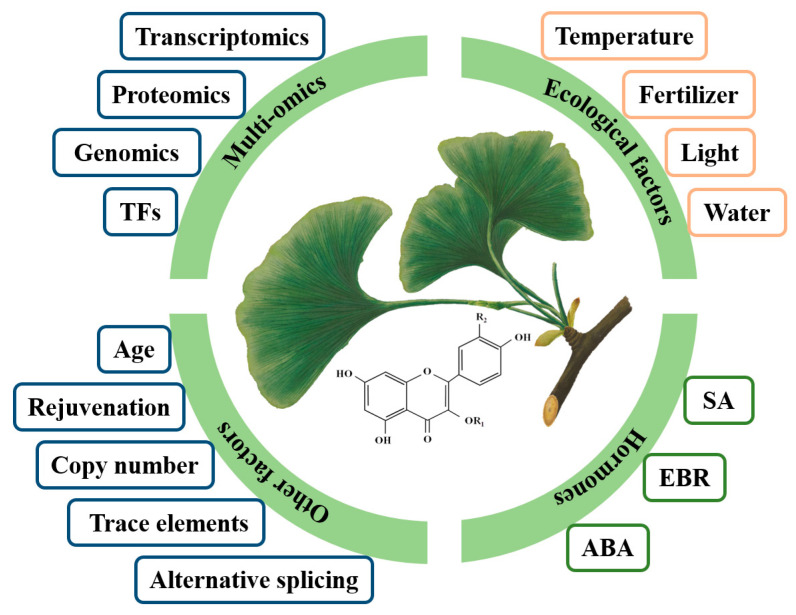
Factors regulating the synthesis and metabolism of flavonoids in ginkgo. TFs, transcription factors; SA, salicylic acid; ABA, abscisic acid; EBR, 2,4-epibrassinolide.

Instead of diving deeper into the biosynthesis pathways of flavonoids in ginkgo, more attention has been given to determining how to increase flavonoid contents. To date, the methods applied to increase flavonoid contents include the optimization of extraction conditions, variety selection, hormone regulation, cultivation condition optimization, and precursor substance addition (Figure 3; [9,62,63,64]). According to practical production considerations, understanding the regulation of flavonoid contents and the relevant mechanism of action is of great significance for the development of high-quality and efficient cultivation measures in ginkgo leaf utilization forests and the enrichment of secondary metabolism theory. Therefore, to support efficient ginkgo cultivation and improve breeding efficiency, it is necessary to devise strategies for increasing active ingredient contents with the aim of finding appropriate measures and simple and efficient techniques that can be used in practical production to enhance the contents of active flavonoid substances in ginkgo in the future.

### 6.1. Hormones

The synthesis of flavonoids is regulated by hormones; thus, exogenous hormone sprays are often used in practical production to increase the flavonoid contents of ginkgo leaves. Foliar spraying is a simple, nontoxic, residue-free, and low-cost technique.

Abscisic acid (ABA) is an isoprenoid-derived phytohormone that is usually associated with plant responses to adversity and shows similarities to flavonoids, effectively improving resistance to stress in plants [65]. The exogenous application of appropriate concentrations of ABA increases flavonoid contents in practice [66]. In ginkgo suspension cell culture, the application of exogenous ABA enhances PAL activity and increases the accumulation of total flavonoids [63]. Exogenous ABA may affect the accumulation of secondary metabolites by regulating endogenous hormone contents and the interhormone balance. Our unpublished results indicate that exogenous ABA can significantly increase flavonoid contents, and this increase is consistent with the increase in ABA content in ginkgo leaves. We hypothesized that ABA is directly responsible for promoting the accumulation of flavonoids at a later stage. This exogenous spraying promotes the accumulation of ginkgo flavonoids by upregulating the expression of key enzyme structural genes such as GbPAL, Gb4CL, GbC4H, and GbF3’H and transcription factors such as GbMYB4 and GbMYB61. The total flavonoid content of T3-generation homozygous transgenic *Arabidopsis* plants with GbMYB4 and GbMYB6 is significantly higher than that of wild-type plants, tentatively confirming the positive regulatory effect of these two genes. Salicylic acid (SA) is a phenolic phytohormone known as a signaling molecule that is capable of stimulating plant defense responses to different biotic and abiotic stresses and can activate a plant’s secondary metabolism [67]. Phytohormone treatment analysis revealed that SA increases the activity and gene expression of PAL enzymes, thereby increasing the total phenolic and flavonoid contents of leaves, with the effect depending on the application concentration [68]. The exogenous spraying of SA can significantly increase the flavonoid content before ginkgo leaves are harvested. The activities of PAL, C4H, 4CL, and antioxidants (peroxidase, superoxide dismutase, and catalase) increase significantly after most SA treatments [62]. Similarly, Ni et al. [69] confirmed that exogenous SA promoted the accumulation of flavonoids in ginkgo leaves and noted that this process involved light; that is, SA-induced flavonoid accumulation requires red and far-red light rather than blue light. The 2,4-epibrassinolide (EBR), a natural sterol, is known as the sixth type of plant hormone. It has a significant comprehensive regulatory function in plant growth, development, and secondary metabolic processes, including the phenylpropanoid pathway [70]. Unpublished results generated by our group indicate that exogenous EBR can promote the expression of the GbMYB59 and GbMYB23 genes and activate the expression of structural genes such as GbPAL, Gb4CL, and GbFLS in the flavonoid metabolic pathway, thus promoting the accumulation of flavonoids.

### 6.2. Ecological Factors

#### 6.2.1. Light

Flavonoids are the main physiologically active components of plants and play an important role in scavenging reactive oxygen species and ensuring the normal growth and development of plants. Changes in external environmental conditions, such as light, water supply, temperature, nutritional status, and especially some degree of adverse conditions, can significantly affect the synthesis and accumulation of flavonoids in ginkgo [71]. Light intensity and duration are important ecological factors driving plant photosynthesis and regulating growth and development, which can influence the primary metabolism and the further accumulation of secondary metabolites [72]. Research on modifying environmental factors to increase the flavonoid content of ginkgo leaves has also been conducted. For example, the use of LED lights to apply red, mixed (red:blue = 1:1), and blue light to treat annual ginkgo seedlings showed that mixed and blue light significantly increased the flavonoid content. In particular, blue light significantly increased the flavonoid yield (0.76-fold increase) and antioxidant capacity, which laid a foundation for the exploitation of light quality in the cultivation of ginkgo leaf utilization forests [73]. The expression of the PAL, CHS, F3H, and FLS genes related to ginkgo flavonoid biosynthesis decreases with an increasing degree of shading [74]. Flavonoid biosynthesis in ginkgo is induced via light irradiation, and the accumulation of flavonoids is positively correlated with the amount of ultraviolet-B (UV-B) radiation. UV-B radiation can increase the contents of flavonoids and their components in ginkgo, but the increasing effect of short-term radiation is limited, and proper prolongation of the radiation time may favor the accumulation of flavonoids [75]. A potential mechanism that has been demonstrated is that UV-B radiation promotes flavonoid accumulation by stimulating the expression of the GbHY5-GbMYB1-GbFLS module in ginkgo leaves [76]. In addition, flavonoids with hydroxyl groups can serve as reducing agents for oxidizing substances, and oxygen radicals accumulated via UV-B radiation can be efficiently scavenged and reduced by them. UV-B radiation also leads to an unbalanced accumulation of polyhydroxy flavonoids in plants, resulting in an increased quercetin/kaempferol ratio [77,78].

#### 6.2.2. Temperature

Environmental temperature alters the biosynthesis pattern of secondary metabolites in ginkgo [39,79]. In general, high temperatures inhibit the biosynthesis of flavonoids and lead to their enzymatic or chemical degradation, while low temperatures can induce the biosynthesis of flavonoids. The expression levels of PAL, C4H, and CHS gradually decrease with increasing temperature, and lower temperatures favor the accumulation of flavonoids in ginkgo leaves and especially the biosynthesis of quercetin derivatives, as quercetin is considered the most effective cryoprotectant of plant leaves [39,79]. In contrast, a high temperature is beneficial for the synthesis of kaempferol derivatives, indicating that different flavonoid components have different optimal synthesis conditions [80]. To some extent, increasing the temperature difference between day and night is beneficial for the synthesis and accumulation of flavonoids. Lower temperatures and soil moisture levels are conducive to the biosynthesis of flavonoids in ginkgo, so silvicultural measures such as establishing leaf utilization plantations in lower-temperature areas and reducing preharvest irrigation can be adopted to increase flavonoid yield [80].

#### 6.2.3. Water and Fertilizer

The ginkgo leaf flavonoid content is significantly and positively correlated with water use efficiency (WUE) and the root-to-shoot ratio and negatively correlated with the total biomass [81]. Alternative partial root zone irrigation and slight drought are beneficial for water management and flavonoid accumulation in ginkgo plantations. In addition, Yu et al. [82] found that partial root zone drying was an effective method for harvesting ginkgo leaves with high concentrations of flavonoids, and structural genes and TFs involved in hormone metabolism and flavonoid biosynthesis pathways play critical roles in regulating flavonoid accumulation. Fertilization can promote the growth of ginkgo and the accumulation of nutrients in leaves as well as hormonal conditions, and the application of fertilization for bud and leaf growth increases leaf numbers and promotes leaf flavonoid accumulation, which increases both ginkgo leaf yields and quality [33,34].

### 6.3. Other Factors

Tree age significantly affects ginkgo leaf quality (active ingredient content, leaf thickness, and leaf mass); thus, the leaves of ginkgo seedlings younger than 7 years are commonly used in production to produce *Ginkgo biloba* extract (GBE) [71]. Analysis at the physiological level has revealed that the biosynthesis of flavonoids may be promoted via jasmonic acid and SA accumulation. Leaf quality decreases with increasing tree age, rejuvenation through coppicing increases leaf biomass and flavonoid accumulation, and the expression of genes involved in flavonoid biosynthesis is upregulated, including CHS, FLS, F3’H, DFR, and LAR [83]. Rejuvenation increases the gibberellin content of ginkgo leaves, and additional research showed that exogenous gibberellin significantly increases the expression of GbCHS and the flavonoid content. This rejuvenation through coppicing changes hormone levels, leaf biomass, and flavonoid contents, which provides a feasible method for increasing ginkgo leaf yield and quality.

Plants require few trace elements, but their roles in plant growth and development are equally important to those of macroelements, and trace element deficiency significantly affects plant growth and development, resulting in decreases in yield and quality. Moderate amounts of exogenous organic selenium and nanoselenium promote the expression of key genes involved in flavonoid biosynthesis and the accumulation of total flavonoids in ginkgo leaves, which is of great significance for improving the medicinal value of GBE [84].

Differences in genome copy number affect transcription and metabolite production in plants. The leaves of grafted haploid ginkgo seedlings are smaller and contain fewer flavonoids than those of diploids, probably due to a smaller number of corresponding regulatory genes and the significant downregulation of genes involved in flavonoid biosynthesis [85]. Moreover, intron retention is the most important type of alternative splicing at different developmental stages of various tissues and plays a key role in regulating flavonoid biosynthesis in ginkgo [86]. The biosynthesis pathways of flavonoids and terpenoids also contain alternative splicing variations, which may affect their synthesis.

## 7. Perspectives

In 1965, the Schwabe company in Germany first launched a ginkgo preparation into the market, introducing the concept of GBE761 into medical practice, and the medicinal value of ginkgo received widespread attention. Injections, liquids, sugar-coated tablets, and sustained-release tablets containing ginkgo leaf extract (GBE761) have good therapeutic effects on heart disease, coronary heart disease, dementia, nervous system diseases, and Alzheimer’s disease. The isolation and identification of physiologically active substances in ginkgo leaves and the development of health foods and medicines with ginkgo extract have become research hot spots. Flavonoids are still obtained from ginkgo leaves. Therefore, it is very important to elaborate the flavonoid biosynthesis pathway and related transcription regulatory factors, as well as ecological factors, to improve flavonoid production in ginkgo to cope with growing global market demand. China has a long history of ginkgo cultivation and is also the origin and main distribution center of ginkgo, harboring abundant germplasm resources with great development and utilization value. These advantages provide unique resources for the research and breeding of ginkgo and can be used to accelerate the diversification of the ginkgo lineage in China [87]. However, a complete industrial chain has not been formed, which will be necessary for the healthy development of the biopharmaceutical industry. There is wasting of resources in many regions, and technology for the development and utilization of extracts is lagging behind that in Western countries. Therefore, conducting research aimed at improving the flavonoid content of ginkgo has become an important direction for its cultivation and breeding.

With the rapid development of sequencing technology [88], many potential key genes have been predicted in the biosynthesis pathway of flavonoids in ginkgo, but verifying the functions of these genes may take a long time. In addition, specific genes closely related to specific flavonoid components are not yet known, and further in-depth mining and exploration are needed for specific flavonoid components. Due to the lack of effective cultivation measures for increasing flavonoid contents on leaf utilization plantations, research on obtaining flavonoids through callus culture is becoming increasingly important. However, there is currently a lack of efficient and stable culture systems for obtaining flavonoids from calli. To meet the industrial production targets for ginkgo flavonoids, a suitable suspension callus culture system needs to be established for ginkgo. Moreover, as a gymnospermous plant, ginkgo has a large genome, a long juvenile stage, and no effective genetic transformation system, which hinders the comprehensive understanding of the functions of genes related to flavonoid biosynthesis. An efficient ginkgo protoplast isolation and transient expression system has recently been established [89], and its reliability was initially verified through subcellular localization, transient overexpression, and protein interactions. However, a stable genetic transformation system still needs to be developed to verify the function of ginkgo flavonoids. Coupled with gene and environmental factor regulation, the precise improvement of ginkgo leaf yields and flavonoid contents and ginkgo germplasm innovation will be achieved in the future.

## Figures and Tables

**Table 1:** Progress in the study of structural genes in ginkgo flavonoid synthesis pathways.

Gene	Function	References
GbPAL	Relative expression levels of GbPAL are significantly correlated with flavonoid contents	[37]
GbC4H	Plays a key role in lignin synthesis, stress, and hormone signaling responses	[38]
Gb4CL	4CL enzyme activity varies under different diurnal temperature combinations	[39]
GbCHS	Homologous to genes in other gymnosperm species; important for flavonoid synthesis	[40]
GbCHI	Activity is positively correlated with total flavonoid levels	[41]
GbF3H	More highly expressed in stems, especially leaves	[42]
GbF3’H1	Overexpression enhances epigallocatechin, gallocatechin, and catechin contents	[35]
GbF3′5′H1	Overexpression improves epicatechin and gallocatechin contents	[33,34]
GbFLS	Recombinant GbFLS protein acts as a bifunctional enzyme in the flavonoid biosynthetic pathway	[43]
GbFLSa	Negatively regulates proanthocyanin biosynthesis	[44]
Three DFRs	GbDFR1 and GbDFR3 catalyze dihydroquercetin transformation to leucocyanidin, while GbDFR2 catalyzes dihydrokaempferol transformation to leucopelargonidin	[45]
GbDFR	Associated with higher anthocyanin contents and darker-colored flowers	[46]
GbDFR6	Overexpression of GbDFR6 changes flowering phenotypes under short-day conditions and increases the contents of many anthocyanins	[47]
GbDFR5	The self-incompatibility-like phenotypes	[48]

## Data Availability

Not applicable.

## References

[1] Jacobowitz J.R., Weng J.K. (2020). Exploring uncharted territories of plant specialized metabolism in the postgenomic era. Annu. Rev. Plant Biol..

[2] Shen N., Wang T., Gan Q., Liu S., Wang L., Jin B. (2022). Plant flavonoids: Classification, distribution, biosynthesis, and antioxidant activity. Food Chem..

[3] Zhou Q., Mu K., Xu M., Ma X., Ni Z., Wang J., Xu L.-A. (2017). Variation in the concentrations of major secondary metabolites in *Ginkgo* leaves from different geographical populations. Forests.

[4] Zhao Y.P., Fan G., Yin P.P., Sun S., Li N., Hong X., Hu G., Zhang H., Zhang F.M., Han J.D. (2019). Resequencing 545 *Ginkgo* genomes across the world reveals the evolutionary history of the living fossil. Nat. Commun..

[5] Guo J., Wu Y., Jiang M., Wu C., Wang G. (2022). An LC–MS-based metabolomic approach provides insights into the metabolite profiles of *Ginkgo biloba* L. at different developmental stages and in various organs. Food Res. Int..

[6] Silva H., Martins F.G. (2022). Cardiovascular activity of *Ginkgo biloba*—An insight from healthy subjects. Biology.

[7] Luo Y., Smith J.V. (2004). Studies on molecular mechanisms of *Ginkgo biloba* extract. Appl. Microbiol. Biotechnol..

[8] Mohammed N.A., Abdou H.M., Tass M.A., Alfwuaires M., Abdel-Moneim A.M., Essawy A.E. (2020). Oral supplements of *Ginkgo biloba* extract alleviate neuroinflammation, oxidative impairments and neurotoxicity in rotenone-induced parkinsonian rats. Curr. Pharm. Biotechnol..

[9] Cheng S.Y., Xu F., Wang Y. (2009). Advances in the study of flavonoids in *Ginkgo biloba* leaves. J. Med. Plants Res..

[10] Liu X.G., Wu S.Q., Li P., Yang H. (2015). Advancement in the chemical analysis and quality control of flavonoid in *Ginkgo biloba*. J. Pharm. Biomed. Anal..

[11] Liu L., Wang Y., Zhang J., Wang S. (2021). Advances in the chemical constituents and chemical analysis of *Ginkgo biloba* leaf, extract, and phytopharmaceuticals. J. Pharm. Biomed. Anal..

[12] Wang H., Shi M., Cao F., Su E. (2022). *Ginkgo biloba* seed exocarp: A waste resource with abundant active substances and other components for potential applications. Food Res. Int..

[13] Boateng I.D. (2022). A review of *Ginkgo biloba* L. seed’s protein; physicochemical properties, bioactivity, and allergic glycoprotein. Food Rev. Int..

[14] Li R., Xia Z., Li B., Tian Y., Zhang G., Li M., Dong J. (2021). Advances in supercritical carbon dioxide extraction of bioactive substances from different parts of *Ginkgo biloba* L.. Molecules.

[15] Hirata B.K.S., Cruz M.M., de Sá R.D.C.C., Farias T.S.M., Machado M.M.F., Bueno A.A., Alonso-Vale M.I.C., Telles M.M. (2019). Potential anti-obesogenic effects of *Ginkgo biloba* observed in epididymal white adipose tissue of obese rats. Front. Endocrinol..

[16] Zhou Z., Zheng S. (2003). The missing link in *Ginkgo* evolution. Nature.

[17] Wang L., Cui J., Jin B., Zhao J., Xu H., Lu Z., Li W., Li X., Li L., Liang E. (2020). Multifeature analyses of vascular cambial cells reveal longevity mechanisms in old *Ginkgo biloba* trees. Proc. Natl. Acad. Sci. USA.

[18] Gong W., Chen C., Dobeš C., Fu C.-X., Koch M.A. (2008). Phylogeography of a living fossil: Pleistocene glaciations forced *Ginkgo biloba* L. (Ginkgoaceae) into two refuge areas in China with limited subsequent postglacial expansion. Mol. Phylogenet. Evol..

[19] Chen Y., Fu C., Wu Z., Xu H., Liu H., Schneider H., Lin J. (2021). *Ginkgo* *biloba*. Trends Genet..

[20] Guan R., Zhao Y., Zhang H., Fan G., Liu X., Zhou W., Shi C., Wang J., Liu W., Liang X. (2016). Draft genome of the living fossil *Ginkgo biloba*. GigaScience.

[21] Guo J., Wu Y., Wang B., Lu Y., Cao F., Wang G. (2016). The effects of fertilization on the growth and physiological characteristics of *Ginkgo biloba* L.. Forests.

[22] Liu Y., Xin H., Zhang Y., Che F., Shen N., Cui Y. (2022). Leaves, seeds and exocarp of *Ginkgo biloba* L. (Ginkgoaceae): A Comprehensive Review of Traditional Uses, phytochemistry, pharmacology, resource utilization and toxicity. J. Ethnopharmacol..

[23] Han X., He B., Xin Y., Xu M., Xu L.A. (2021). Full-length sequencing of *Ginkgo biloba* L. reveals the synthesis of terpenoids during seed development. Ind. Crops Prod..

[24] Wang H.Y., Zhang Y.Q. (2019). The main active constituents and detoxification process of *Ginkgo biloba* seeds and their potential use in functional health foods. J. Food Compos. Anal..

[25] Gong G., Guan Y.Y., Zhang Z.L., Rahman K., Wang S.J., Zhou S., Luan X., Zhang H. (2020). Isorhamnetin: A review of pharmacological effects. Biomed. Pharmacother..

[26] van Beek T.A. (2002). Chemical analysis of *Ginkgo biloba* leaves and extracts. J. Chromatogr. A.

[27] Klomsakul P., Aiumsubtub A., Chalopagorn P. (2022). Evaluation of antioxidant activities and tyrosinase inhibitory effects of *Ginkgo biloba* tea extract. Sci. World J..

[28] Liao Z., Cheng L., Li X., Zhang M., Wang S., Huo R. (2020). Meta-analysis of *Ginkgo biloba* Preparation for the Treatment of Alzheimer’s Disease. Clin. Neuropharmacol..

[29] Dong H.L., Lin S., Wu Q.L., Su R.X., Wu Z.L., Dong H.Y., Li H.L., Zhang W.D. (2020). A new bilobalide isomer and two cis-coumaroylated flavonol glycosides from *Ginkgo biloba* leaves. Fitoterapia.

[30] Šamec D., Karalija E., Dahija S., Hassan S.T.S. (2022). Biflavonoids: Important contributions to the health benefits of *Ginkgo* (*Ginkgo biloba* L.). Plants.

[31] Wang Z., Gan S., Sun W., Chen Z. (2022). Widely targeted metabolomics analysis reveals the differences of nonvolatile compounds in oolong tea in different production areas. Foods.

[32] Liu S., Meng Z., Zhang H., Chu Y., Qiu Y., Jin B., Wang L. (2022). Identification and characterization of thirteen gene families involved in flavonoid biosynthesis in *Ginkgo biloba*. Ind. Crops Prod..

[33] Wu D., Feng J., Lai M., Ouyang J., Liao D., Yu W., Wang G., Cao F., Jacobs D.F., Zeng S. (2020). Combined application of bud and leaf growth fertilizer improves leaf flavonoids yield of *Ginkgo biloba*. Ind. Crops Prod..

[34] Wu Y., Wang T., Xin Y., Wang G., Xu L.A. (2020). Overexpression of GbF3′5′H1 provides a potential to improve the content of epicatechin and gallocatechin. Molecules.

[35] Wu Y., Wang T., Xin Y., Wang G., Xu L.A. (2020). Overexpression of the GbF3′H1 gene enhanced the epigallocatechin, gallocatechin, and catechin contents in transgenic Populus. J. Agric. Food Chem..

[36] Wu Y., Guo J., Zhou Q., Xin Y., Wang G., Xu L.-a. (2018). De novo transcriptome analysis revealed genes involved in flavonoid biosynthesis, transport and regulation in *Ginkgo biloba*. Ind. Crops Prod..

[37] Xu F., Cai R., Cheng S., Du H., Wang Y. (2008). Molecular cloning, characterization and expression of phenylalanine ammonia-lyase gene from *Ginkgo biloba*. Afr. J. Biotechnol..

[38] Cheng S., Yan J., Meng X., Zhang W., Liao Y., Ye J., Xu F. (2018). Characterization and expression patterns of a cinnamate-4-hydroxylase gene involved in lignin biosynthesis and in response to various stresses and hormonal treatments in *Ginkgo biloba*. Acta Physiol. Plant..

[39] Guo J., Zhou X., Wang T., Wang G., Cao F. (2020). Regulation of flavonoid metabolism in *Ginkgo* leaves in response to different day-night temperature combinations. Plant Physiol. Biochem..

[40] Pang Y., Shen G.A., Liu C., Liu X., Tan F., Sun X., Tang K. (2004). Molecular cloning and sequence analysis of a novel chalcone synthase cDNA from *Ginkgo biloba*. DNA Seq..

[41] Cheng H., Li L., Cheng S., Cao F., Wang Y., Yuan H. (2011). Molecular cloning and function assay of a chalcone isomerase gene (GbCHI) from *Ginkgo biloba*. Plant Cell Rep..

[42] Shen G., Pang Y., Wu W., Deng Z., Zhao L., Cao Y., Sun X., Tang K. (2006). Cloning and characterization of a flavanone 3-hydroxylase gene from *Ginkgo biloba*. Biosci. Rep..

[43] Xu F., Li L., Zhang W., Cheng H., Sun N., Cheng S., Wang Y. (2012). Isolation, characterization, and function analysis of a flavonol synthase gene from *Ginkgo biloba*. Mol. Biol. Rep..

[44] Guo J., Wu Y., Wang T., Xin Y., Wang G., Zhou Q., Xu L.-A. (2023). GbFLSa overexpression negatively regulates proanthocyanin biosynthesis. Front. Plant Sci..

[45] Hua C., Linling L., Shuiyuan C., Fuliang C., Feng X., Honghui Y., Conghua W. (2013). Molecular cloning and characterization of three genes encoding dihydroflavonol-4-reductase from *Ginkgo biloba* in anthocyanin biosynthetic pathway. PLoS ONE.

[46] Ni J., Ruan R., Wang L., Jiang Z., Gu X., Chen L., Xu M. (2020). Functional and correlation analyses of dihydroflavonol-4-reductase genes indicate their roles in regulating anthocyanin changes in *Ginkgo biloba*. Ind. Crops Prod..

[47] Ni J., Zhang N., Zhan Y., Ding K., Qi P., Wang X., Ding W., Xu M. (2022). Transgenic tobacco plant overexpressing *Ginkgo* dihydroflavonol 4-reductase gene GbDFR6 exhibits multiple developmental defects. Front. Plant Sci..

[48] Zhang N., Zhan Y., Ding K., Wang L., Qi P., Ding W., Xu M., Ni J. (2023). Overexpression of the *Ginkgo biloba* dihydroflavonol 4-reductase gene GbDFR6 results in the self-incompatibility-like phenotypes in transgenic tobacco. Plant Signal. Behav..

[49] Khairul-Anuar M.-A., Mazumdar P., Othman R.Y., Harikrishna J.A. (2022). DhMYB22 and DhMYB60 regulate pigment intensity and floral organ shape in Dendrobium hybrid. Ann. Bot..

[50] Wang L., Zhao J., Zhang M., Li W., Luo K., Lu Z., Zhang C., Jin B. (2015). Identification and characterization of microRNA expression in *Ginkgo biloba* L. leaves. Tree Genet. Genomes.

[51] Liu S., Wang L., Cao M., Pang S., Li W., Kato-Noguchi H., Jin B., Wang L. (2020). Identification and characterization of long non-coding RNAs regulating flavonoid biosynthesis in *Ginkgo biloba* leaves. Ind. Crops Prod..

[52] Deng C., Wu Y., Lv X., Li J., Liu Y., Du G., Chen J., Liu L. (2022). Refactoring transcription factors for metabolic engineering. Biotechnol. Adv..

[53] Zhao L., Gao L., Wang H., Chen X., Wang Y., Yang H., Wei C., Wan X., Xia T. (2013). The R2R3-MYB, bHLH, WD40, and related transcription factors in flavonoid biosynthesis. Funct. Integr. Genom..

[54] Yang X., Zhou T., Wang M., Li T., Wang G., Fu F.F., Cao F. (2021). Systematic investigation and expression profiles of the GbR2R3-MYB transcription factor family in *Ginkgo* (*Ginkgo biloba* L.). Int. J. Biol. Macromol..

[55] Xu F., Ning Y., Zhang W., Liao Y., Li L., Cheng H., Cheng S. (2013). An R2R3-MYB transcription factor as a negative regulator of the flavonoid biosynthesis pathway in *Ginkgo biloba*. Funct. Integr. Genom..

[56] Zhang W., Xu F., Cheng S., Liao Y. (2018). Characterization and functional analysis of a MYB gene (GbMYBFL) related to flavonoid accumulation in *Ginkgo biloba*. Genes Genom..

[57] Su X., Xia Y., Jiang W., Shen G., Pang Y. (2020). GbMYBR1 from *Ginkgo biloba* represses phenylpropanoid biosynthesis and trichome development in Arabidopsis. Planta.

[58] Zhou X., Liao Y., Kim S.-U., Chen Z., Nie G., Cheng S., Ye J., Xu F. (2020). Genome-wide identification and characterization of bHLH family genes from *Ginkgo biloba*. Sci. Rep..

[59] Zheng J., Liao Y., Xu F., Zhou X., Ye J., Fu M., Liu X., Cao Z., Zhang W. (2021). Genome-wide identification of WD40 superfamily genes and prediction of WD40 gene of flavonoid-related genes in *Ginkgo biloba*. Not. Bot. Horti Agrobot. Cluj Napoca.

[60] Xin Y., Wu Y., Han X., Xu L.-a. (2021). Overexpression of the *Ginkgo biloba* WD40 gene GbLWD1-like improves salt tolerance in transgenic Populus. Plant Sci..

[61] Han H., Xu F., Li Y., Yu L., Fu M., Liao Y., Yang X., Zhang W., Ye J. (2021). Genome-wide characterization of bZIP gene family identifies potential members involved in flavonoids biosynthesis in *Ginkgo biloba* L.. Sci. Rep..

[62] Guo Y., Feng Y., Fu F.F., El-Kassaby Y.A., Wang T., Wang G. (2021). Eliciting increased flavonoids content in *Ginkgo biloba* leaves through exogenous salicylic acid and methyl jasmonate treatments. Can. J. For. Res..

[63] Hao G., Du X., Zhao F., Ji H. (2010). Fungal endophytes-induced abscisic acid is required for flavonoid accumulation in suspension cells of *Ginkgo biloba*. Biotechnol. Lett..

[64] Ni J., Dong L., Jiang Z., Yang X., Sun Z., Li J., Wu Y., Xu M. (2018). Salicylic acid-induced flavonoid accumulation in *Ginkgo biloba* leaves is dependent on red and far-red light. Ind. Crops Prod..

[65] Chen K., Li G.J., Bressan R.A., Song C.P., Zhu J.K., Zhao Y. (2020). Abscisic acid dynamics, signaling, and functions in plants. J. Integr. Plant Biol..

[66] Li G., Zhao J., Qin B., Yin Y., An W., Mu Z., Cao Y. (2019). ABA mediates development-dependent anthocyanin biosynthesis and fruit coloration in Lycium plants. BMC Plant Biol..

[67] Janda M., Ruelland E. (2015). Magical mystery tour: Salicylic acid signalling. Environ. Exp. Bot..

[68] Tajik S., Zarinkamar F., Soltani B.M., Nazari M. (2019). Induction of phenolic and flavonoid compounds in leaves of saffron (*Crocus sativus* L.) by salicylic acid. Sci. Hortic..

[69] Ni J., Hao J., Jiang Z., Zhan X., Dong L., Yang X., Sun Z., Xu W., Wang Z., Xu M. (2017). NaCl induces flavonoid biosynthesis through a putative novel pathway in post-harvest *Ginkgo* leaves. Front. Plant Sci..

[70] Zhang L., Ahammed G.J., Li X., Wei J.-P., Li Y., Yan P., Zhang L.P., Han W.Y. (2018). Exogenous brassinosteroid enhances plant defense against colletotrichum gloeosporioides by activating phenylpropanoid pathway in *Camellia sinensis* L.. J. Plant Growth Regul..

[71] Wang Q., Jiang Y., Mao X., Yu W., Lu J., Wang L. (2022). Integration of morphological, physiological, cytological, metabolome and transcriptome analyses reveal age inhibited accumulation of flavonoid biosynthesis in *Ginkgo biloba* leaves. Ind. Crops Prod..

[72] Jaakola L., Hohtola A. (2010). Effect of latitude on flavonoid biosynthesis in plants. Plant Cell Environ..

[73] Wang G., Zhang L., Wang G., Cao F. (2022). Growth and flavonol accumulation of *Ginkgo biloba* leaves affected by red and blue light. Ind. Crops Prod..

[74] Xu Y., Wang G., Cao F., Zhu C., Wang G., El-Kassaby Y.A. (2014). Light intensity affects the growth and flavonol biosynthesis of *Ginkgo* (*Ginkgo biloba* L.). New For..

[75] Zhao B., Wang L., Pang S., Jia Z., Wang L., Li W., Jin B. (2020). UV-B promotes flavonoid synthesis in *Ginkgo biloba* leaves. Ind. Crops Prod..

[76] Liu S., Gu X., Jiang Y., Wang L., Xiao N., Chen Y., Jin B., Wang L., Li W. (2023). UV-B promotes flavonoid biosynthesis in *Ginkgo biloba* by inducing the GbHY5-GbMYB1-GbFLS module. Hortic. Res..

[77] Schreiner M., Mewis I., Huyskens-Keil S., Jansen M.A.K., Zrenner R., Winkler J.B., O’Brien N., Krumbein A. (2012). UV-B-induced secondary plant metabolites-potential benefits for plant and human health. Crit. Rev. Plant Sci..

[78] Sun M., Gu X., Fu H., Zhang L., Chen R., Cui L., Zheng L., Zhang D., Tian J. (2010). Change of secondary metabolites in leaves of *Ginkgo biloba* L. in response to UV-B induction. Innov. Food Sci. Emerg. Technol..

[79] Wang G., Cao F., Wang G., El-Kassaby Y.A. (2015). Role of temperature and soil moisture conditions on flavonoid production and biosynthesis-related genes in *Ginkgo* (*Ginkgo biloba* L.) leaves. Nat. Prod. Chem. Res..

[80] Wang G., Cao F., Chang L., Guo X., Wang J. (2014). Temperature has more effects than soil moisture on biosynthesis of flavonoids in *Ginkgo* (*Ginkgo biloba* L.) leaves. New For..

[81] Wang L., Shi H., Wu J., Cao F. (2016). Alternative partial root-zone irrigation enhances leaf flavonoid accumulation and water use efficiency of *Ginkgo biloba*. New For..

[82] Yu W., Liu H., Luo J., Zhang S., Xiang P., Wang W., Cai J., Lu Z., Zhou Z., Hu J. (2022). Partial root-zone simulated drought induces greater flavonoid accumulation than full root-zone simulated water deficiency in the leaves of *Ginkgo biloba*. Environ. Exp. Bot..

[83] Lu Z., Zhu L., Lu J., Shen N., Wang L., Liu S., Wang Q., Yu W., Kato-Noguchi H., Li W. (2022). Rejuvenation increases leaf biomass and flavonoid accumulation in *Ginkgo biloba*. Hortic. Res..

[84] Deng K., Li L., Li L., Xu F., Yuan H., Zha S., Xiao X., Yu J., Cheng S., Cheng H. (2022). Molecular Mechanism of Selenium Affecting the Synthesis of Flavonoids in *G. biloba* Leaves. Plant Mol. Biol. Report..

[85] Hu Y., Zhang Y., Šmarda P., Bureš P., Guo Q. (2023). Transcriptome and proteome associated analysis of flavonoid metabolism in haploid *Ginkgo biloba*. Int. J. Biol. Macromol..

[86] He B., Han X., Liu H., Bu M., Cui P., Xu L.-A. (2022). Deciphering alternative splicing patterns in multiple tissues of *Ginkgo biloba* important secondary metabolites. Ind. Crops Prod..

[87] Šmarda P., Horová L., Knápek O., Dieck H., Dieck M., Ražná K., Hrubík P., Orlóci L., Papp L., Veselá K. (2018). Multiple haploids, triploids, and tetraploids found in modern-day “living fossil” *Ginkgo biloba*. Hortic. Res..

[88] Liu H., Wang X., Wang G., Cui P., Wu S., Ai C., Hu N., Li A., He B., Shao X. (2021). The nearly complete genome of *Ginkgo biloba* illuminates gymnosperm evolution. Nat. Plants.

[89] Han X., Rong H., Feng Y., Xin Y., Luan X., Zhou Q., Xu M., Xu L.-A. (2023). Protoplast isolation and transient transformation system for *Ginkgo biloba* L.. Front. Plant Sci..

